# The Relationship between Thermal Comfort and Light Intensity with Sleep Quality and Eye Tiredness in Shift Work Nurses

**DOI:** 10.1155/2013/639184

**Published:** 2013-02-13

**Authors:** Hiva Azmoon, Habibollah Dehghan, Jafar Akbari, Shiva Souri

**Affiliations:** Occupational Health Engineering Department, School of Health, Isfahan University of Medical Sciences, Isfahan 81746, Iran

## Abstract

Environmental conditions such as lighting and thermal comfort are influencing factors on sleep quality and visual tiredness. The purpose of this study was the determination of the relationship between thermal comfort and light intensity with the sleep quality and eye fatigue in shift nurses. *Method.* This cross-sectional research was conducted on 82 shift-work personnel of 18 nursing workstations in Isfahan Al-Zahra Hospital, Iran, in 2012. Heat stress monitoring (WBGT) and photometer (Hagner Model) were used for measuring the thermal conditions and illumination intensity, respectively. To measure the sleep quality, visual tiredness, and thermal comfort, Pittsburg sleep quality index, eye fatigue questionnaire, and thermal comfort questionnaire were used, respectively. The data were analyzed with descriptive statistics, Student's *t*-test, and Pearson correlation. *Results.* Correlation between thermal comfort which was perceived from the self-reporting of people with eye tiredness was −0.38 (*P* = 0.002). Pearson correlation between thermal comfort and sleep quality showed a positive and direct relationship (*r* = 0.241, *P* = 0.33) but the correlation between thermal comfort, which was perceived from the self-reporting of shift nurses, and WBGT index was a weak relationship (*r* = 0.019). *Conclusion.* Based on the obtained findings, it can be concluded that a defect in environmental conditions such as thermal conditions and light intensity and also lack of appropriate managerial plan for night shift-work nurses are destructive and negative factors for the physical and mental health of this group of practitioners.

## 1. Introduction

Nursing is a profession which, because of its stressful nature, is being faced with unforeseen situations, shifting, and organizational and personal factors, and these agents affected the mental and physical health of nurses. Different studies have reported that, in comparison with the mental and physical health of people of our country, the mental and physical health of this group of society is in a lower level [1]. Altered circadian rhythm of the autonomic regulation can have important implications on the psychological and medical problems related to shift work. For example, increase in job stress and deterioration in mood or cognitive performance were seen in shift workers [2, 3], which may be associated with altered autonomic function [4, 5]. 

Some studies have reported that interns working 12 h consecutive night shifts demonstrated a reduction in visual memory capacity over the night shift [6]. Other studies have reported that sleep-deprived residents demonstrated impaired alertness or concentration [7, 8], were less efficient, and made more clinical errors [9]. Some studies have reported that working acute night shifts reduced the performance of monotonous tasks and increased sleepiness [10].

Findings of a research in electrical industries of Tehran in 2004 denoted that the inadequate level of illumination intensity causes headache and neurosis [11]. The results of a study showed that the level of lighting affected practitioners' performance and their eye fatigue [12]. In a research on improving nurse performance with appropriate design of light, which was done by Kamalii and Abbas in 2011, it was found that with a appropriate lighting design, the performance of nurses was improved, and their fault rate was diminished [13].

Among the other variables of workplace, thermal comfort can be mentioned. The thermal comfort feeling of an individuals depends on their thermal stability. Thermal stability also depends on different parameters such as physical activity rate, garment, and atmospheric conditions (dry bulb temperature, radiant temperature, air moisture, and air speed). Thermal comfort based on ANSI/ASHRAE-SS 1981 Standard is described as a condition in which, a human being is mentally satisfied with thermal condition [14, 15]. Mental satisfaction from thermal condition of workplace is one of the issues that should be considered in hospitals, since hospital indoor environment, because of climate conditions specific to the care of patients, can be uncomfortable for nurses. Hence, they should be noticed seriously.

Among the problems of night shift nurses, sleep quality can be named. In the past few years, nursing has been in the spotlight, because of its work injuries. In a research which was done on 2372 nurses in Baltimore, USA, it was shown that more than 1/4 of nurses work about 12 hours a day and more than half of them have more than one job; therefore, they become extremely tired and this causes work injuries [16]. There is no doubt that there is an extreme pressure and accuracy in nursing, that is why all the factors that may increase human faults should be studied. Among these factors, we can mention sleep quality. Sleep is one of the vital needs of human beings. Adequate sleep improves the performance of neurons and the immunity of body and has a dramatic influence on the learning process, growth, and evolution [17]. Another study result shows that people with sleeping problems receive 7 times more injuries compared with people who have enough sleep. 

The most important factors that can influence level of comfort of people in a building can be divided in to two sets: human factors and environmental factors [18]. Environmental factors can be evaluated by heat stress monitoring, thermal comfort questionnaires, and illumination intensity measuring. Buchanan et al. showed that there is a linear relationship between each pharmacist's error rate and that pharmacist's corresponding daily prescription workload for three illumination levels (45, 102, and 146 foot candle), so that the rate of prescription-dispensing errors was associated with the level of illumination [19]. Beauchemin and Hays reported that patients in sunny rooms had an average stay of 16.6 days compared to 19.5 days for those in dull rooms, a difference of 2.6 days [20].

Nurses, in comparison with others, experience disorders like misappropriate sleep quality, pressure, and stress [1]. Based on a research which was done about effects of different shifts of female nurses, long period night shifts increase cancer risk in women, in comparison with women who do not work at night shifts [21, 22]. Also in a case-control research, they studied the work shifts and cancer risk of Danish nurses. They found out that the nurses who work in turning shifts after midnight are more likely to contract cancer in comparison with the nurses who work in a fixed shift in daylight [17]. In a reviewing research under the name of “women's sleep, their health and their illness,” different aspects of inadequate sleep and sleep disorders were studied among women. The results showed that in addition to environmental parameters and work conditions, inadequate sleep and sleep quality are influenced by physical, mental, and hormonal health [23]. In another research on one effect of work-sleep cycle of physical health between shift nurses, the results determined that the night shift nurses contract physical illnesses more than nurses who work in daylight [24]. In another research, they studied the sleep quality of night shift and fixed shift nurses. The results showed that there is a magnificent difference between the sleep qualities of the two mentioned groups. The fixed shift nurses enjoyed better sleep quality [25]. Hence, this study was done with the purpose of determining the relationship between environmental conditions of workplace such as light intensity in night shift nurses, heat stress, and thermal comfort with eye fatigue and sleep quality.

## 2. Method

### 2.1. Subjects

In this study, 88 (74 men and 14 women) of 300 nurses of night shift nurses were chosen voluntarily and simple randomly; their average (standard deviation) of age, height, BMI, and work history were 30.75 (6.42) years, 74.04 (13.15) kg, 174.65 (8.38) cm, 24.17 (3.27) kg/m^2^, and 9.7 (3.5) years, respectively. The inclusion criteria were having at least 1-year work record, not having cardiovascular or respiratory diseases, and not receiving any sedatives. Exclusion criteria were not willing to cooperate or not filling out the questionnaires completely (two of the participants were removed).

## 3. Procedure

This cross-sectional study was done on night shift nurses of Isfahan Al-Zahra Hospital, in 16 nursing workstation, in 2012. In the present study, illumination intensity was measured by luxmeter (Hugner model), visual fatigue was determined by eye fatigue questionnaire that is comprising 15 questions, and answers have been designed on 0–10 Likert range. Alpha coefficient of the questionnaire was 0.755 [26], and sleep quality was also measured by Pittsburg sleep quality index (PSQI) that sensitivity and reliability of PSQI waere 89.6% and 86.5%, respectively, [27]. This questionnaire yields 7 scores for the individual's overall description of sleep quality, the period of sleeping, the period of effective sleep, effective sleep, sleep disorders, dose of receiving opiate, daily performance disorders scales, and one overall score. The score of each questionnaire has been considered from 0 to 3 in which 0, 1, 2, and 3 imply normal condition, presence of a little, mean, and intense problems, respectively [28]. The thermal conditions including dry bulb temperature, wet bulb temperature, globe temperature, and WBGT index were measured by Heat Stress Monitor (Casella Microtherm, Ireland). Data collecting carried out at between 9 PM and 1 AM Data analyzing was done by descriptive statistics, Pearson correlation analysis, and regression analysis.

## 4. Results

**Figure 1:**
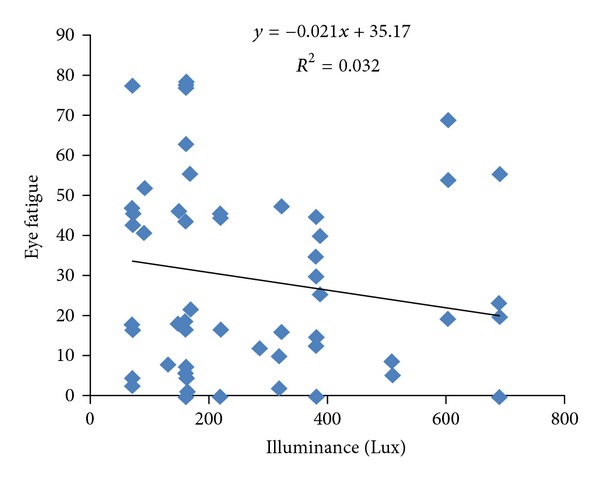
Scatter plot between illuminance and eye fatigue in shift work nurses.

**Figure 2:**
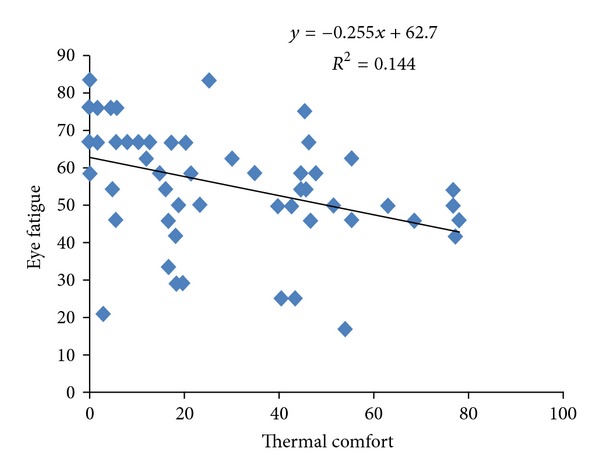
Scatter plot between thermal comfort and eye fatigue in shift work nurse.

**Figure 3:**
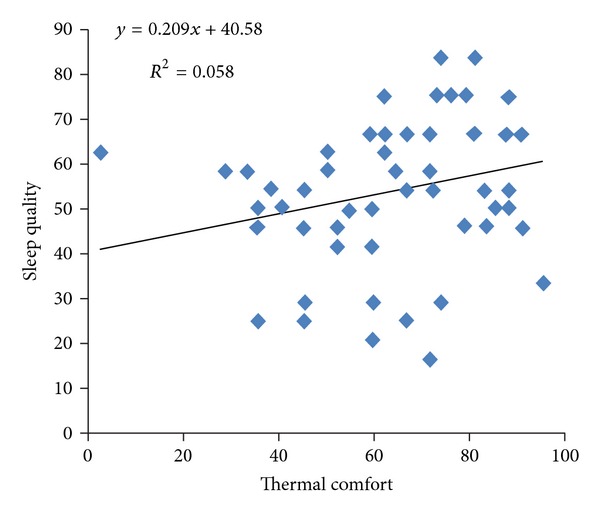
Scatter plot between thermal comfort and sleep quality in shift work nurses.

**Figure 4:**
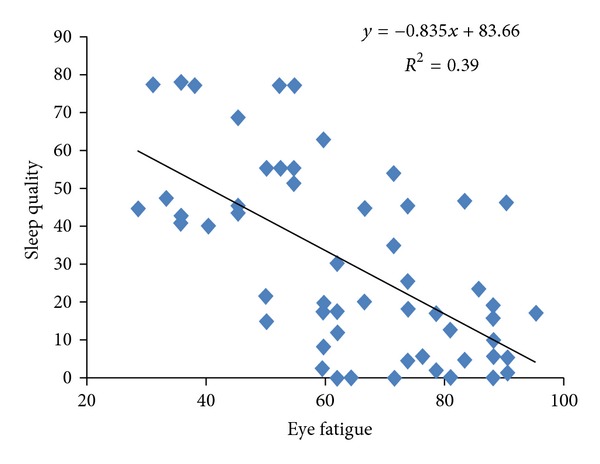
Scatter plot between eye fatigue and sleep quality in shift work nurses.

The mean (standard deviation) of WBGT indicator, for all workstations of hospital, was 20.67(0.55)°C (range 19.60–22.20°C). The mean (standard deviation) of light intensity for all hospital's workstations was 296 lux (range 70–680 lux). The mean (standard deviation) of thermal comfort score was 53.47 (17.15) (range 16.67–83.3). The mean (standard deviation) of eye fatigue score among these people was 29.06 (24.28). The results of Pearson correlation between illumination intensity and the other variables represent that eye fatigue has a reverse relation with illumination intensity (*r* = −0.179) (Figure 1). Pearson correlation between illumination intensity and sleep quality has a weak relation (*r* = 0.017). The yielded results of Pearson correlation between thermal comfort and WBGT index and the other variables represent that reverse. Pearson correlation between thermal comfort and WBGT indicator has a weak relation (*r* = 0.019). Pearson correlation between thermal comfort and eye fatigue shows a significant and reverse relation (*P* = 0.002, *r* = −0.38) (Figure 2). Pearson correlation between thermal comfort and sleep quality shows a positive and significant relation (*P* = 0.033, *r* = 0.24) (Figure 3). Pearson correlation between eye fatigue and sleep quality indicates a positive and significant relation (*P* < 0.001, *r* = −0.66) (Figure 4). Among the other studied relations, we can mention that the Pearson correlation between sleep quality variables, eye fatigue, and thermal comfort with age variable, which the only significant relation was the Pearson correlation between age and sleep quality. The relation in the latter was reverse and significant (*P* = 0.021, *r* = 0.26). Finally, the Pearson correlation between age and thermal comfort indicates a reverse and weak relation (*r* = −0.065). The correlation between age and eye fatigue has shown a weak relation (*r* = 0.138).

## 5. Discussion

Appropriate light intensity is essential and important in performance of work activities. As the results of the present study indicate too, light intensity and eye fatigue have a reverse relationship, and this indicates that with the increase of light intensity, the eye fatigue of night shift nurses diminished, and also statistical analysis of relations (Pearson correlation) in the present study shows that with the increase of light levels intensity, thermal comfort and sleep quality of nurses (even very little) has improved. These results are the same as the result of Hansen and Stevens 2012 study. In the mentioned study, with improving the light intensity of night nurses' workplace, their conciseness, performance, and comfort improved [17]. 

Sleep disorder is one of the most important issues among shift nurses, especially night shift nurses. High stress and disarranged shifts are important effective factors on sleep disorders and sleep quality [13]. The results of the current research, like the others which have been conducted before, indicate that night shift nurses (participants in this research) suffer from sleep disorders [28]. A research in 1992 is one of the researches with the same results, which describes the poor sleep quality of shift nurses very clearly. One of its reasons could be lacking an adequate sleep model, and this inadequate and disordered sleep model may have originated from personal and environmental factors such as inadequate and inappropriate light intensity in work environment (which affects eye fatigue). The results of the present study have shown such relationships, for example, a positive relation between light intensity and sleep quality [11]. 

Among the important and interesting results of this study is the correlation between eye fatigue and sleep quality which is a reverse and significant relation. This relation indicates that the less the level of eye fatigue decreases, the more the level of their sleep quality increases. This is because of the following reason. When people in workplace face inadequate level of light intensity, they try to make up this defect by pressuring on their eye muscles and continue to their activities and complete their tasks. This leads to eye tiredness and frequent headaches which can be a negative effective factor on sleep quality itself [12]. This defect can be fixed by improving the light intensity of the environment. 

Thermal condition is one of the other environmental factors influencing physical and mental health state of people. Pearson correlation results between eye fatigue and sleep quality and nurses' thermal comfort indicate that the increase in thermal comfort contributes to the decrease in eye fatigue of nurses and improvement in their sleep quality; therefore, it can be inferred that we can decrease eye fatigue of shift nurses and improve their sleep quality by improving thermal condition of work environment. 

Finally, it is presumed that the relationship between eye tiredness and sleep quality can be a mutual relationship. Level of light intensity can be an effective factor in removing eye tiredness and improving sleep quality of people, especially our shift nurses. Disorder in circadian rhythm as a consequence of inadequate level of daylight in work environment is the other reason for decrease in sleep quality of nurses which can lead to a decrease in the production of sleep hormone [29].

## 6. Conclusion

 Sleep quality and eye fatigue in night shift, especially in night shift nurses, can readily under the different factors including mental-psychological state, occupational and social factors, and environmental factors such as light intensity and thermal condition. Therefore, based on results of the present study, it can be said that we can diminish eye tiredness of these hard-working practitioners and improve their sleep quality by improving the workplace conditions such as light intensity and thermal condition. Thus, conducting the same and more comprehensive research is necessary and desirable to determine the relationship between other physical variables of work environment and diverse variables related to physical and mental health of this group of people more accurately.
